# Enhanced reproducibility of SADI web service workflows with Galaxy and Docker

**DOI:** 10.1186/s13742-015-0092-3

**Published:** 2015-12-03

**Authors:** Mikel Egaña Aranguren, Mark D. Wilkinson

**Affiliations:** 1 Genomic Resources, Department of Genetics, Physical Anthropology and Animal Physiology, Faculty of Science and Technology, University of Basque Country (UPV/EHU), Sarriena auzoa z/g, Leioa – Bilbo, 48940 Spain; 2 Biological Informatics, Centre for Plant Biotechnology and Genomics (CBGP), Technical University of Madrid (UPM), Campus of Montegancedo, Pozuelo de Alarcón, 28223 Spain; 3 Eurohelp Consulting, 48011 Maximo Aguirre 18, Bilbo, Spain

**Keywords:** Semantic Web, RDF, SADI, Web service, Workflow, Galaxy, Docker, Reproducibility

## Abstract

**Background:**

Semantic Web technologies have been widely applied in the life sciences, for example by data providers such as OpenLifeData and through web services frameworks such as SADI. The recently reported OpenLifeData2SADI project offers access to the vast OpenLifeData data store through SADI services.

**Findings:**

This article describes how to merge data retrieved from OpenLifeData2SADI with other SADI services using the Galaxy bioinformatics analysis platform, thus making this semantic data more amenable to complex analyses. This is demonstrated using a working example, which is made distributable and reproducible through a Docker image that includes SADI tools, along with the data and workflows that constitute the demonstration.

**Conclusions:**

The combination of Galaxy and Docker offers a solution for faithfully reproducing and sharing complex data retrieval and analysis workflows based on the SADI Semantic web service design patterns.

## Background

**Fig. 1:**
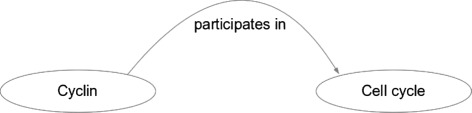
RDF triple. The predicate (‘participates in’) goes from subject (‘Cyclin’) to object (‘Cell cycle’)
- *SPARQL Protocol and RDF Query Language (SPARQL).* SPARQL is a query language to extract data from RDF graphs [4].
- *Web Ontology Language (OWL).* OWL is a knowledge representation language for making assertions about the interpretation of data using axioms that facilitate the application of automated reasoning (e.g. ‘A protein participates in at least one biological process’) [5]. Therefore, OWL is used to create ontologies that codify the consensus of a community about their knowledge domain. In an OWL ontology, there are several different types of entities: individuals are the actual instances of data (e.g. ‘Cyclin’, ‘Mark’, or ‘Madrid’); properties link individuals to one another (e.g. ‘Mark lives in Madrid’); and classes are combinations of logical axioms and properties that make the distinction between one kind of individual and another (e.g. ‘Protein’ or ‘Human’). Finally, individuals are assigned to a class based on the logical match between their properties, and on the class definition: for example, ‘Mark’ is a ‘Human’, because it lives in a city, and ‘Cyclin’ is a ‘Protein’, because it participates in at least one biological process.

The Semantic Web is a ‘third-generation’ web in which information is published directly as data, in machine-processable formats [1]. With the Semantic Web, the web becomes a ‘universal database’, rather than the collection of documents it has traditionally been. As a consequence, on the Semantic Web information is retrieved by directly querying the data, rather than parsing documents, leading to more accurate results. Furthermore, automatic agents can browse the data, finding information and generating new hypotheses that would be difficult to generate for a human user alone. Though the Semantic Web is not yet pervasive, it has been deployed extensively in the life sciences, where Semantic Web technologies are used to integrate data from different resources with disparate schemas [2]. The Semantic Web is made possible through a set of standards proposed by the WWW Consortium, including the following:

- *Resource Description Framework (RDF).* RDF is a machine-readable data representation language based on the ‘triple’, that is, data is codified in a subject–predicate–object structure (e.g. ‘Cyclin participates in Cell cycle’, Fig. 1), in which the predicate and object (‘participates in’ and ‘Cell cycle’, respectively) describe a property of the subject (‘Cyclin’) [3]. In RDF, it is common for entities to be the object of one triple and the subject of another triple. Thus triples can be connected to one another. A collection of connected triples is called a graph, and graphs are commonly stored in triple stores to facilitate their query and exploration, where the triples tore is akin to a database.

The backbone of the Semantic Web is the fact that Uniform Resource Identifiers (URIs) [6] are used to identify all entities (OWL classes, instances, and properties, and RDF subjects, predicates, and objects). This allows one to refer to entities located in external resources on the web: for example, in an RDF triple, the subject might be indicated by a URI from one resource and the predicate and object by a URI from a different resource.

The most widely used principles for publishing Semantic Web data are those that have emerged from the Linked Data community. The core Linked Data principles are (adapted from [7, 8]):

1. Identify every data item (entity or relationship) with a URI.
2. Make those URIs Hypertext Transfer Protocol (HTTP) resolvable, that is, when the URI is requested a document containing information about the entity can be obtained.
3. Provide the information using an open formatting standard when an entity is requested by HTTP. The format provided should be determined by HTTP content negotiation between the client and the server (e.g. RDF for an automatic agent, or Hypertext Markup Language (HTML) for a human user), so that the entity and its representations are decoupled. Importantly, the RDF format should always be available.
4. Ensure, to the greatest extent possible, that the information provided by URI resolution contains typed relations to other entities, so that the agent can traverse those relations to discover new information, analogously to how humans browse the web.

Linked Data has demonstrated clear value as a means of data publication in a machine-readable and web-resolvable fashion, opening up new possibilities for data discovery and integration [9]. As a result, significant life sciences data providers have implemented Linked Data solutions for their resources, including UniProt [10], EBI RDF [11], and OpenLifeData [12], each of which contributes to the growth of the Linked Open Data cloud [13].

In addition to data representation, Semantic Web standards have also been applied to analytical tools, for example through the creation of Semantic Web services. The Semantic Automated Discovery and Integration (SADI) design pattern [14] is unique among the Semantic Web service initiatives in that SADI presumes that all data is (or eventually will be) Linked Data, and therefore SADI services process Linked Data natively. SADI makes it possible to retrieve data in exactly the same way, from every service, without the overhead that other web service technologies demand: with SADI services, RDF data is passed to a service, verbatim and without any message scaffolding, by HTTP POST; the response is the same data ‘decorated’ with new RDF triples, making integration and consumption of the data (even with other tools) straightforward. Recently, the OpenLifeData2SADI project has implemented the SADI principles to expose the more than 6 billion linked data points in the OpenLifeData warehouse, providing automatically discoverable access to each data point via one of several thousand SADI services [8].

This article shows how to combine OpenLifeData2SADI data retrieval services with SADI analytical services, using off-the-shelf tools from the popular Galaxy bioinformatics platform [15], provided as a Docker image. Additionally, a worked example is provided as a ready-to-use exemplar of data and an appropriate workflow, making the procedure trivially reproducible computationally (with Docker) and functionally (with Galaxy). This approach provides multiple advantages, not the least of which is that this easy reproducibility allows the potential for third parties to explore a wide variety of modifications.

## Findings

### Technical elements

#### SADI services

SADI is a set of design patterns based on Semantic Web standards for providing web services. It does not define any new technology or schema, nor even a message-passing infrastructure. Instead, it uses off-the-shelf, well-established technologies and formats (URI, RDF, and OWL) to provide all of its discoverability and interoperability features. In a SADI service, the data the service consumes is defined by an OWL class: the client uses automated reasoning to infer whether the RDF it possesses is a member of that OWL class, and if so, the client may simply HTTP POST the RDF to the service. Once the service has processed the input, it creates an output Linked Data graph by connecting the input RDF subject node to additional triples generated by the analytical algorithm of the service. Effectively, SADI services produce new chains of Linked Data [8].

#### OpenLifeData2SADI

The Bio2RDF project captures existing data from numerous life sciences providers and republishes it with normalized URIs and Linked Data support [16]. In turn, the OpenLifeData project reformats Bio2RDF data and enhances its content negotiation functionality. On top of this, OpenLifeData2SADI offers access to OpenLifeData through a set of automatically generated SADI services [8]. This semantically rich OpenLifeData can be discovered and retrieved in a consistent and predictable manner, by a machine, simply by calling the appropriate SADI service. Importantly, the retrieved RDF can then be easily integrated with other Linked Data from any source.

#### Galaxy

**Fig. 2:**
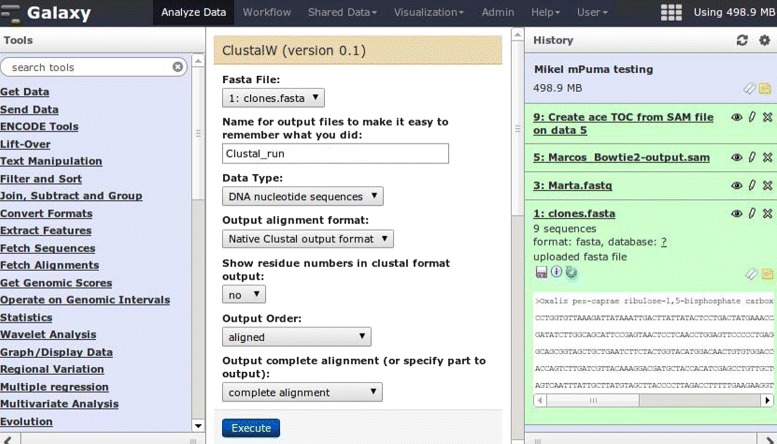
The Galaxy main interface (reproduced with permission from [19]) Galaxy is a web server with several different interfaces: ‘Analyze data’, ‘Workflow’, ‘Shared data’, etc. The main interface, ‘Analyze data’ (shown here), is where data is analyzed with different tools (left column) and a history is recorded (right column), so that workflows can be extracted (they will appear in the ‘Workflow’ interface). In ‘Shared data’, histories, data, and workflows can be shared between users and/or published

Galaxy is a web server that offers an infrastructure within which biologists can analyze data via a consistent web interface (Fig. 2). A history of the tasks performed is stored so that workflows with common steps can be extracted from the history and rerun independently. The most common bioinformatics tools are already included in the Galaxy distribution, and new tools can be created by simply wrapping command line executables in Galaxy-compliant eXtensible Markup Language (XML) files. There are many public Galaxy servers, and Galaxy can also be installed privately.

#### Docker

Docker [17] is a virtualization engine and runtime system. The key difference from a virtual machine is that a Docker image shares resources with the host operating system (OS), making images lighter (in the case where the host is a GNU/Linux system). Containers can be run, with the Docker engine, from predefined images. Docker Hub [18], a repository of images, is also available, so a developer can build an image with the desired computational environment (OS, libraries, configuration), software, and data, starting from a pre-existing image (e.g. Ubuntu 14.04), which is then deployed back to the repository. Then anyone can retrieve this customized image and run it as a container, including the new software, without configuration or installation.

### Worked example

#### Merging OpenLifeData2SADI and SADI services in a single workflow

**Fig. 3:**
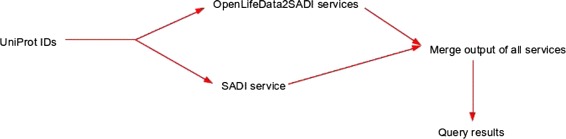
Conceptual representation of example workflow. The workflow starts from a set of UniProt identifiers and obtains information from OpenLifeData SADI services and regular SADI services. The output is merged into a single dataset and queried

**Fig. 4:**
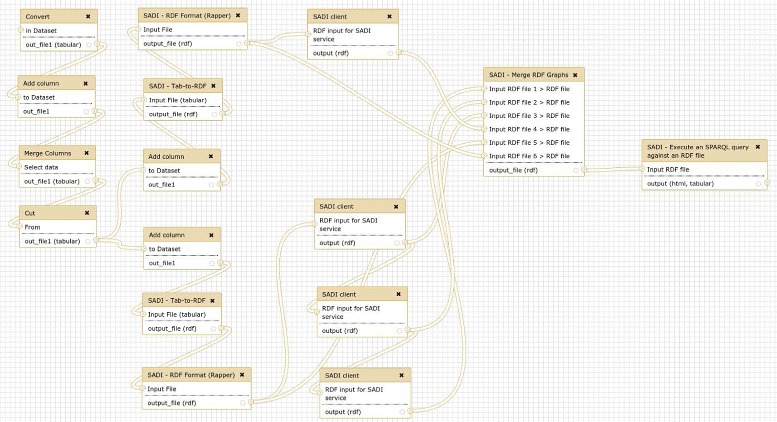
Screenshot of the actual Galaxy workflow that implements the general idea described in Fig. 3. The workflow executes two groups of SADI services, and therefore the input UniProt identifiers must be converted into two RDF datasets, but the first steps of the process are shared (from ‘Convert’ to ‘Cut’). Then the appropriate RDF triple is added to each UniProt identifier (after ‘cut’, from ‘Add column’ to ‘RDF Format’, twice) and SADI services are called (‘SADI client’). The output of the SADI services and the input RDF are merged into a single graph (‘Merge RDF Graphs’), which is then queried (‘Execute an SPARQL query against an RDF file’), producing the results in Tab Separated Values (TSV) format and HTML format

An example workflow shows how OpenLifeData2SADI and the archetypal SADI analytical services can be merged (Figs. 3 and 4). This workflow, while novel, builds upon the workflows presented in [8, 19].

The workflow answers the following question: Given a set of UniProt proteins, which ones are related to PubMed abstracts containing the term ‘brain’, and what are their Kyoto Encyclopedia of Genes and Genomes (KEGG) [20] entries? The workflow starts from a simple list of UniProt identifiers, and retrieves different datasets from a regular SADI service (to obtain KEGG entries) and a chain of three OpenLifeData2SADI services (to obtain PubMed abstracts). The results are then merged and queried to obtain the KEGG entries of proteins that are related to PubMed abstracts that contain the term. The workflow involves five steps, explained as follows.

##### 1. Obtain a list of UniProt identifiers of interest.

This can be done, for example, by simply uploading the list from a local computer or importing it directly to Galaxy from Biomart [21]:

**Figure:**
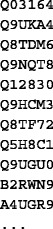

##### 2. Convert the input to RDF.

For data to be consumed by the SADI services, it needs to be converted to RDF. Additionally, an rdf:type triple must be added to each identifier that asserts the OWL input class of each SADI service, producing two different inputs from the same list of UniProt identifiers. The triple<Uniprot identifier> rdf:type http://purl.oclc.org/SADI/LSRN/UniProt_ Record is added for the service to retrieve KEGG entries (getKEGGIDFromUniProt), resulting in the following RDF:

**Figure:**
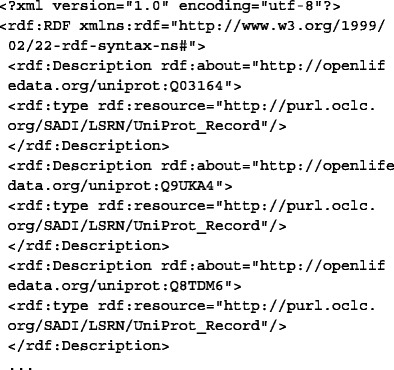

The triple<Uniprot identifier> rdf:type http://openlifedata.org/uniprot_vocabulary: Resourceis added for OpenLifeData2SADI services, resulting in the following RDF:

**Figure:**
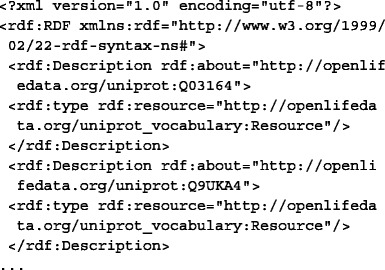

##### 3. Send the appropriate input to services.

Each of the RDF inputs is sent to the appropriate OpenLifeData2SADI service (three services in a row) and to getKEGGIDFromUniProt.

##### 4. Merge the outputs and the inputs into a single RDF graph.

Because SADI services track their data inputs by way of the incoming subject URIs (new predicates and objects are added to the input URIs, while maintaining the URIs for the output), the outputs of the services are immediately merged with the inputs into a single graph, with no additional action required.

##### 5. Query the merged graph with SPARQL.

**Fig. 5:**
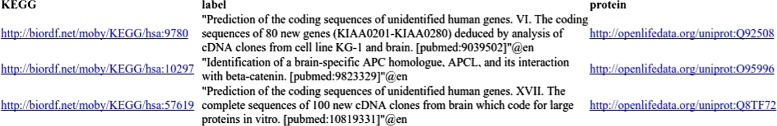
The result of the workflow is a list of PubMed abstracts containing the term ‘Brain’, with related proteins and KEGG entries (‘@en’ refers to the fact that the abstract is in english language). The result can be displayed as HTML, for browsing the actual resources in their web pages, or TSV, for downstream analysis in Galaxy

In this case, the UniProt entries from the input set that are mentioned in a PubMed abstract containing the term ‘brain’ and their respective KEGG entries are retrieved with the following query (Fig. 5):

**Figure:**
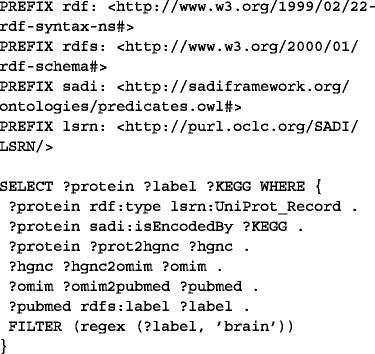

#### Reproducing the workflow through Galaxy and Docker

The Docker image contains the developed tools, dependencies, and running environment [22]. The image is based on the base image Ubuntu:14.04, and it installs, through apt-get, all the necessary libraries. The image also copies, from the path in which it is built, the SADI client and related tools. All the Docker commands that build the image can be found in the following Docker file:

**Figure:**
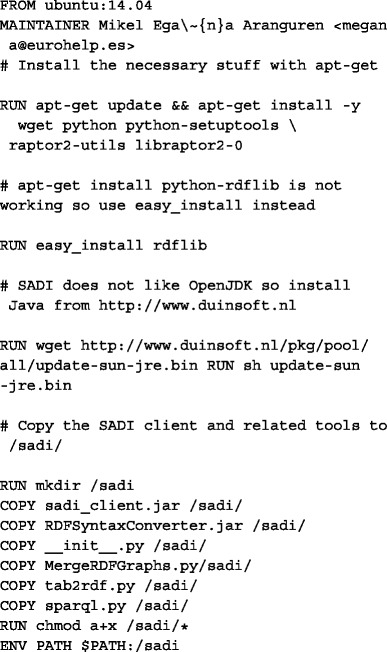

The image can be built by pasting the above instructions in a Docker file and runing docker build, but more importantly, the image can be obtained from the Docker central registry by docker pull (assuming a GNU/Linux system with the Docker engine installed):

$ docker pull mikeleganaaranguren/sadi:v6

The Galaxy tools needed to invoke the executables of the Docker image are:

- SADI client: a SADI client for synchronous SADI services (adapted from [19]).
- RDFSyntaxConverter: a tool to convert between different RDF syntaxes, including from RDF to TSV files (adapted from [19]).
- MergeRDFgraphs: a tool to merge different RDF graphs into one (adapted from [19]).
- SPARQLGalaxy: a tool to perform SPARQL queries against RDF files (adapted from [19]).
- Rapper: a tool to convert RDF files to different syntaxes.
- Tab2rdf: a tool to produce RDF files from TSV files.

**Fig. 6:**
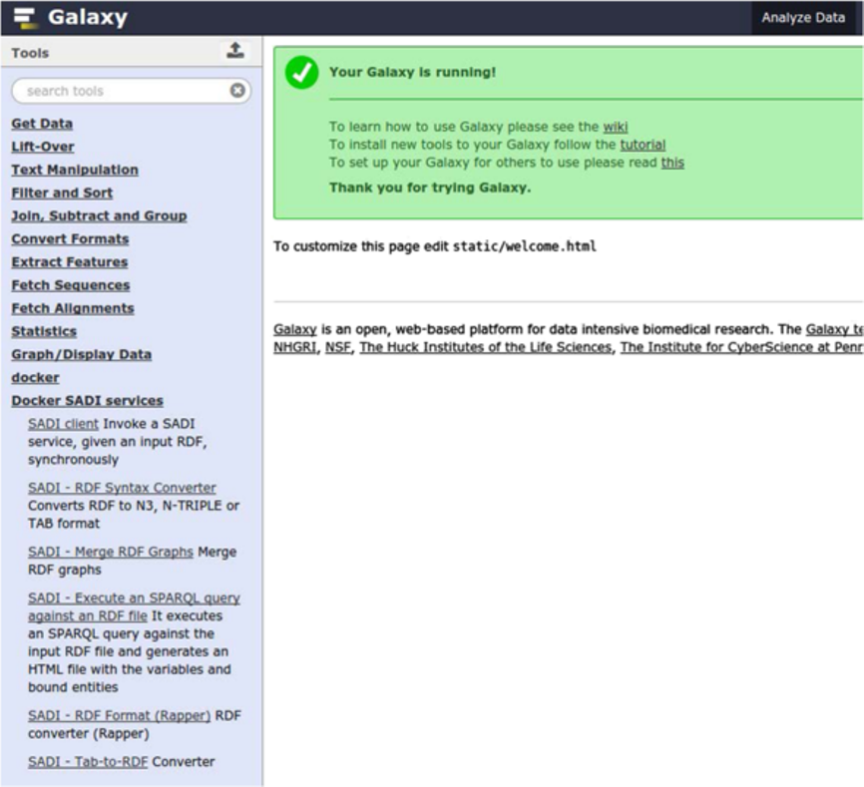
Galaxy server interface showing SADI-Docker tools. The tools are available on the left column of the Galaxy interface, under ‘Docker SADI services’: clicking on any of them will show a menu that can be used to invoke the tool

**Fig. 7:**
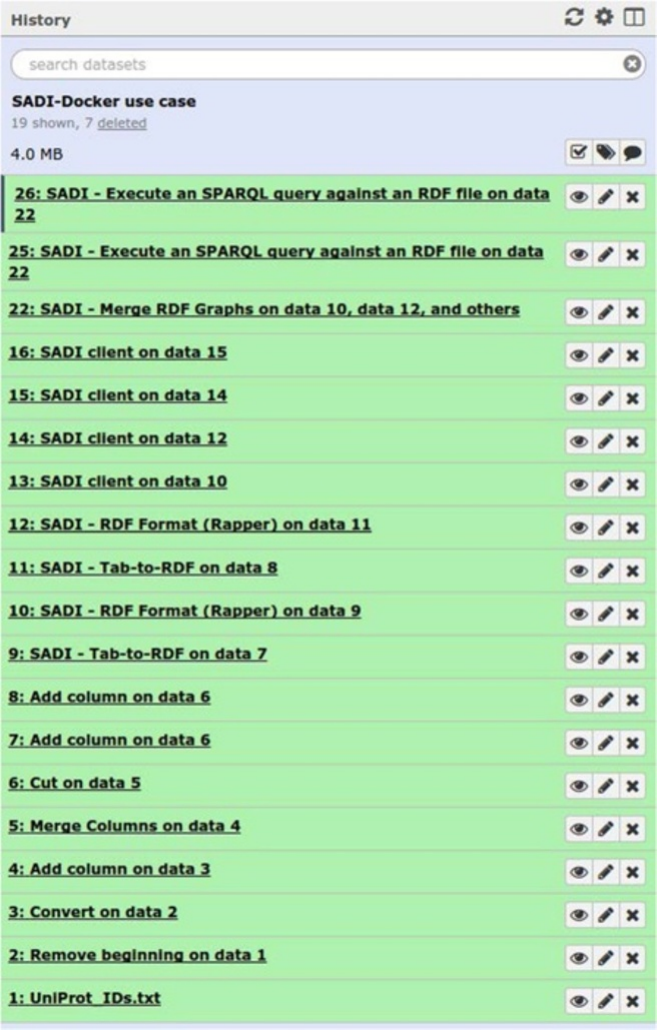
Galaxy server interface showing history after workflow execution. The history is available on the right column of the Galaxy interface, and each line represents a step on the workflow (the green color means that the step has successfully finished). Each step can be re-run independently

These tools are available in the Galaxy Toolshed as a single repository [23]. The workflow is also available in the Toolshed [24] and in the SADI-Docker GitHub repository [25]. Figure 6 shows the SADI-Docker tools after installation, and Fig. 7 shows the result of successfully executing the use case workflow.

To run the workflow, the following steps should be followed (detailed instructions can be found at the SADI-Docker repository in GitHub):

1. Install the Docker image in the local Docker repository, by pulling it.
2. Install Galaxy.
3. Install the SADI-Docker Galaxy tools (from the Toolshed or manually).
4. Upload the test dataset provided in the SADI-Docker GitHub repository, with the UniProt IDs, to Galaxy.
5. Import the workflow (from the Toolshed or manually) and run it, providing the test dataset as the input for the first step of the workflow.

### Discussion

#### Data integration and manipulation through RDF and SADI

Accessing Linked Data is typically accomplished by retrieving the content of a URL or by composing SPARQL CONSTRUCT queries over a static triples tore. SADI therefore adds considerable power to the current Semantic Web infrastructure by adding analytics and dynamic content to this milieu. Because SADI has no API (beyond standard HTTP GET and POST), it is easily integrated into other Linked Data tools and environments. Moreover, accessing and chaining SADI services simply involves passing RDF data from one tool to the next. The output from these chains of services is an unbroken chain of RDF that can be queried using SPARQL, as with any other Linked Data.

The RDF data model used by SADI is easily constructed from other, often non-standardized, formats such as TSV by a simple mapping process. Similarly, the output from SADI services can be transformed into non-RDF formats using custom mapping tools or, for example, standard XML stylesheet transforms. Therefore creating Galaxy tools that work with SADI data and services is relatively straightforward, and many tools are available ‘off the shelf’.

Finally, because SADI services work natively with RDF data, many (indeed most) of the URIs contained in the output of the services are also URLs, i.e. they not only identify but also locate entities on the web. As a consequence, much of the final dataset is ‘clickable’, sending the user directly into the source dataset’s website (e.g. OpenLifeData or KEGG URLs; see Fig. 5) – a user-friendly way of enabling further exploration of results.

#### Reproducibility with Galaxy and Docker

Computational reproducibility is becoming an important consideration in the life sciences [26, 27]. This use case demonstrates a procedure by which Linked Data retrieval and analysis workflows can be documented and published in a completely reproducible fashion, by implementing reproducibility at two levels:

1. *Virtualization of the computational environment (OS) through Docker.* Docker allows encapsulation of a complex environment with all the necessary data and software [28]. In this case, an Ubuntu 14.04 image is shipped, with SADI and its dependencies installed, which means that the user need only log into the Galaxy instance that executes Docker images.
2. *Reproducibility of previously performed analyses through Galaxy.* Galaxy is a suitable environment for executing SADI services in a reproducible manner, because it provides an infrastructure in which the workflow management, history, and provenance, and data storage are pre-established [29]. This means that any SADI-based analysis, if performed in a Galaxy instance, is easily reproducible. For example, the same workflow can be repeated every time OpenLifeData is updated and the workflow can be modified and/or fused with other workflows.

## Conclusions

Using a SADI-Docker image invoked by Galaxy, data manipulation and analysis processes can be described, executed, published, shared, and reused with complete transparency, and with little or no configuration required. Because of the API-free, straightforward invocation mechanism for SADI services, workflows can easily be modified to accommodate new data or different contexts. This then provides a tool for the distribution of case implementations in multiplatform environments. The use of the Galaxy interface additionally provides a single foundation for integration of services, the construction of RDF graphs, and their subsequent querying. The worked example presented here provides a tangible illustration of the use of Semantic Web constructs and standards for the extraction of new information from disparate, independent services, in a completely reproducible manner.

## Availability and requirements

- Project name: SADI-Docker-Galaxy.
- Project home page: http://github.com/mikel-egana-aranguren/SADI-Docker-Galaxy.
- Operating system: any OS, as long as Docker is installed.
- Programming languages: Go, Java, and Python.
- Other requirements: Docker, Galaxy.
- License: General Public License (GPL).

## Availability of supporting data

The data supporting the results of this article are available as a workflow in the Galaxy Toolshed [24] and an input dataset in the project repository [30]. Snapshots are also stored in the GigaScience GigaDB repository [31].

## Abbreviations

HTML: hypertext markup language
HTTP: hypertext transfer protocol
KEGG: kyoto encyclopedia of genes and genomes
OS: operating system
OWL: web ontology language
RDF: resource description framework
SADI: semantic automated discovery and integration
SPARQL: SPARQL protocol and RDF query language
TSV: tab separated values
URI: uniform resource identifier
XML: eXtensible markup language
